# Effects of Web-Based Symptom Monitoring Program on Symptom Interference, Physical Activity, and Emergency Department Readmissions in Patients With Pre-Capillary Pulmonary Hypertension: Randomized Controlled Trial

**DOI:** 10.2196/76883

**Published:** 2025-09-15

**Authors:** Miao-Yi Chen, Chun-Hsien Wu, Shu-Meng Cheng, Ting-Yu Chen, Chieh-Yu Liu, Yun-Ju Wang, Chi-Wen Kao, Jen-Jiuan Liaw

**Affiliations:** 1 Graduate Institute of Medical Sciences, College of Medicine, National Defense Medical University, Taipei, Taiwan; 2 Division of Urology, Department of Surgery, Tri-Service General Hospital, National Defense Medical University, Taipei, Taiwan; 3 Division of Cardiology, Department of Internal Medicine, Tri-Service General Hospital, National Defense Medical University, Taipei, Taiwan; 4 Department of Nursing, Chang Gung University of Science and Technology, Chiayi, Taiwan; 5 Institute of Community Health Care, National Yang Ming Chiao Tung University, Taipei, Taiwan; 6 Department of Nursing, Tri-Service General Hospital, National Defense Medical University, Taipei, Taiwan; 7 School of Nursing, College of Nursing, National Defense Medical University, No 161, Sec 6, Minchuan E Rd, Neihu District, Taipei, 11490, Taiwan, 886 87923100 ext 18764

**Keywords:** web-based symptom monitoring program, pre-capillary pulmonary hypertension, 6-minute walk test, randomized controlled trial, emergency department, dyspnea

## Abstract

**Background:**

Pre-capillary pulmonary hypertension (PH) is a progressive and incurable disease characterized by high morbidity, frequent emergency department (ED) visits, and persistently poor survival despite targeted therapies. Web-based symptom monitoring programs offer a promising, non-invasive approach to support self-management and enable the early detection of decompensation.

**Objective:**

This study aimed to evaluate the effects of a web-based symptom-monitoring program on symptom interference, physical activity, and ED readmission in patients with pre-capillary PH.

**Methods:**

This parallel-group, single-blind, randomized controlled trial recruited patients with precapillary PH from a cardiology outpatient department in northern Taiwan. Patients were included if they had been diagnosed with pulmonary arterial hypertension or chronic thromboembolic pulmonary hypertension and had a mean pulmonary arterial pressure of >20 mmHg confirmed via right heart catheterization. Participants were randomized into 2 groups: an intervention group (n=26), which received a 6-month symptom monitoring program delivered via a web-based application, or a control group (n=25), which received standard care. Outcomes were assessed by comparing measures at baseline (enrollment) with measures at 3, 6, and 9 months postintervention. The primary outcome was symptom interference, and the secondary outcomes included physical activity based on the 6-minute walk test, hemodynamic data, and readmissions to the ED. Changes in baseline measurements were analyzed using generalized estimating equations.

**Results:**

The mean age of the 51 participants was 59.6 (SD 13.6) years. Most patients were diagnosed with connective tissue disease-associated pulmonary arterial hypertension (39.2%), with a mean duration of PH since diagnosis of 3.38 (SD 2.55) years. The patient characteristics did not differ significantly between the groups. Compared with the control group, the intervention group experienced greater reduction in symptom interference, and over the 9-month intervention period, the intervention group showed a significantly greater improvement in the 6-minute walk test, with the distance increasing by an average of 9.8 meters every 3 months (*β*=9.81; 95% CI 0.93 to 18.70; *P*=.03). Additionally, generalized estimating equations analysis demonstrated that, compared to the control group, the intervention group had a 65% decrease (*β*=−1.03; OR=0.35; *P*=.04) in the likelihood of ED readmissions for every 3-month interval during the 9-month study period.

**Conclusions:**

A web-based symptom monitoring program effectively reduced symptom interference, improved physical activity, and decreased ED visits in patients with pre-capillary PH.

## Introduction

Pulmonary hypertension (PH) affects approximately 1% of the global population, highlighting its significant public health impact [1,2]. The World Health Organization (WHO) classifies PH into 5 groups, with 2 prominent subtypes characterized by pre-capillary PH: pulmonary arterial hypertension (PAH) and chronic thromboembolic pulmonary hypertension (CTEPH) [3]. Both conditions are rare but may share similar vascular remodeling mechanisms [4]. Untreated, mean pulmonary arterial pressure (mPAP) progressively increases, leading to worsening symptoms and eventual right heart failure, the leading cause of mortality in these patients [3,4].

Persistent dyspnea in patients with PH is a signal of disease exacerbation and can severely limit activity, worsening the overall prognosis [5,6]. Syncope and arrhythmias, as well as complications from PH-specific medications, can increase the need for visits to the emergency department (ED) [7]. Older patients with PH admitted to the ED in the United States are more likely to be admitted to the hospital and experience higher inpatient mortality [8].

Despite the availability of targeted medications for various types of PH, survival rates have not significantly improved, and PH remains incurable [9]. Managing PH is challenging because of the complexity of the disease. However, patients who are proactive and actively engage in self-care activities are better able to manage PH, cope with non-specific symptoms, improve physical performance, and maintain quality of life [10-12]. An effective partnership between patients and health care professionals has been demonstrated to be essential for optimal disease management [3].

The technology of telemedicine allows patients to monitor and manage chronic symptoms associated with severe illnesses or chronic diseases remotely, which can reduce symptoms and hospitalizations [13,14]. Providing strategies for recognizing and managing symptoms related to PH can improve outcomes and reduce mortality [3,10]. Active engagement can not only improve symptom management but also reduce psychological stress [11,15]. Web-based education can support this process by offering detailed management strategies and high-quality information promptly for effective self-monitoring of PH in a home environment [16].

Patients with PH are often challenged by the need for medication titration, stabilization of vital signs, or mitigation of adverse drug effects, which can require prompt medical interventions [17,18]. Remote self-monitoring of weight gain, hypotension, syncope, and dyspnea has been shown to be effective for early detection of critical symptoms, which can enable timely interventions and reduce hospital readmissions [19]. Invasive techniques provide early warnings of decompensation, helping to decrease readmission rates [20]. However, non-invasive methods of monitoring via smartphone and smartwatch applications can track health metrics like heart rate, blood pressure, and oxygen saturation, leading to improvements in outcomes of physical activity, such as increased walking distance, and reductions in hospitalizations [21-23].

There is limited research on whether remote monitoring of symptoms using integrated telemedicine benefits patients with pre-capillary PH. Therefore, this randomized controlled trial aimed to investigate whether a 6-month web-based symptom monitoring intervention reduced symptom interference in patients with pre-capillary PH compared with patients receiving only usual care. Our primary hypothesis was that participants who received the intervention would engage in more strategies for managing symptoms and experience a greater reduction in symptom interference compared with participants in the control group. Therefore, our secondary hypotheses were that, compared to the control group, the intervention group would (1) demonstrate improvements in physical activity as measured by the 6-minute walk test (6MWT), (2) show echocardiographic improvements and lower levels of N-terminal pro-brain natriuretic peptide (NT-pro-BNP), and (3) experience fewer ED readmission events.

## Methods

### Study Design and Participants

This randomized controlled trial with parallel-group design, single blinding, and repeated measures was conducted at one medical center in Taiwan specializing in PAH. The study aimed to assess the effects of a 6-month web-based symptom monitoring program intervention for patients with pre-capillary PH compared with a control group receiving usual standard care.

Patients were eligible to participate if they had PAH or non-operable or recurrent CTEPH and a diagnosis of pre-capillary PH (mPAP >20 mmHg), pulmonary arterial wedge pressure ≤15 mmHg, and pulmonary vascular resistance >2 Wood units via right heart catheterization (RHC) based on 2022 ESC/ERS (European Society of Cardiology/European Respiratory Society) PH guidelines [3]. The inclusion criteria for the study were as follows: (1) age 20 years and older; (2) with PH diagnosed with PAH or non-operable or recurrent CTEPH; (3) diagnosis via RHC; (4) in World Health Organization functional classification for severity of pulmonary hypertension (WHO-FC) I- IV [3]; (5) fluency in Chinese, with the ability to understand and follow instructions; and (6) capable of using a smartphone or computer. The exclusion criteria were (1) non-pre-capillary PH, (2) combined post- and pre-capillary PH, (3) not diagnosed with PH via catheterization, (4) a diagnosis of any mental disability, and (5) inability to use the internet. All eligible participants provided signed informed consent before enrollment. The participants were randomly assigned to either the intervention or control group using a block randomization procedure with sealed envelopes. Randomization was conducted using the web-based Research Randomizer, stratified by sex and by the World Health Organization (WHO) functional classification of PH.

### Sample Size and Power Analysis

Prior to initiating the study, we conducted an a priori power analysis using G*Power software (version 3.1) [24]. Given the rare and severe nature of pre-capillary PH and the potential recruitment uncertainties due to the ongoing COVID-19 pandemic, we conservatively assumed a medium effect size (Cohen *d*=0.5), consistent with widely accepted conventions [25] and previous digital health studies in similar patient populations [23]. Based on this effect size, with an alpha level of .05 and a power greater than 0.8, we initially calculated the required sample size of 53 participants. Considering the high risk of attrition and mortality inherent in this patient group under the pandemic, the attrition rate was estimated to be about 45%‐50%, giving an expected sample size of 100 at the trial registration. Before the randomization, however, 4 patients died. Despite our efforts, only 51 eligible participants were ultimately enrolled and completed the study. Prolonged recruitment periods were deemed impractical. Thus, our research team decided to stop the recruitment of the new patients due to the severe pandemic. To confirm the adequacy of our sample size, we performed a post hoc power analysis using R software (version 4.3.0; R Foundation for Statistical Computing) [26]. With a final sample size of 51 participants, an alpha of .05, and a medium effect size (*f*=0.25, equivalent to Cohen *d*=0.5), statistical power remained above 0.8. This confirms that our sample size was sufficient to support the validity of our findings.

### Ethical Considerations

This study was approved by the hospital’s Institutional Review Board (IRB) (IRB: C202105020) and adhered to the principles of the Declaration of Helsinki and Good Clinical Practice guidelines. A research nurse explained the study procedures to each participant before obtaining signed informed consent. The study was registered with ClinicalTrials.gov (Identifier: NCT05908019) prior to patient enrollment and reported according to the Consolidated Standards of Reporting Trials [27]. Owing to the high attrition and morbidity and uncontrolled pandemic in Taiwan in 2022 and 2023, our research team predicted that the attrition rate could achieve 45%‐50%, so we set the expected sample size of 100 when we registered the trial. The estimated sample size was about 50‐55 patients with power 0.8. In this study, we finalized 51 participants to safeguard the statistical power and validity of the study and minimize additional loss to follow-up under the pandemic.

Participants were assured that their privacy and confidentiality would be strictly protected. All collected data were anonymized and stored on secure, password-protected servers accessible only to authorized study personnel. Identifiable information was removed prior to data analysis. Participants were informed of their right to withdraw from the study at any time without penalty or need to provide a reason. No financial compensation was provided for participation; however, participants received full access to the web-based platform and educational materials at no cost.

### Procedures

#### The Intervention

Participants randomized to the intervention group received a 6-month web-based symptom monitoring program and 3 months following, which was developed to facilitate proactive, daily self-management for patients with pre-capillary PH. This secure, interactive web-based platform enabled participants to report symptoms, monitor vital signs, and access comprehensive support from their health care team.

Participants received 1 week of a structured orientation program. They received instruction in the use of blood pressure monitors and OSTAR finger-type pulse oximeters (Taiwan) for daily monitoring of systolic and diastolic blood pressure, heart rate, and peripheral oxygen saturation (SpO₂) (ideally performed each morning), and also received information on how to recognize symptoms guided by WHO-FC criteria. Each participant was then provided with unique login credentials for secure access to the system, and the remainder of the orientation included instructions on how to navigate the web-based platform.

The web-based monitoring system included 4 features: daily record keeping, feedback about abnormal readings for monitors, patient education and self-care resources, and direct access to care team members. Participants were provided with a detailed, step-by-step instruction booklet for easy reference at home. A screenshot of the login page and the opening page of the monitoring platform is shown in the overview of the monitoring platform in Multimedia Appendix 1. Details of the 4 core features of the platform are provided below.

**Figure 1.**
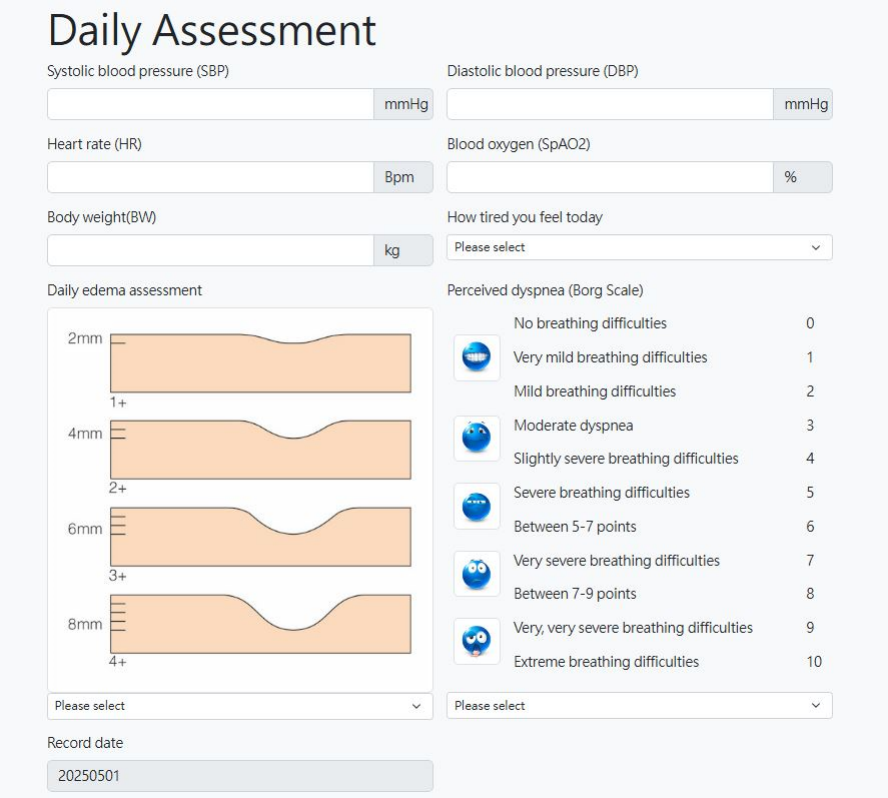
Web-based platform for daily assessments.

The record-keeping section contained baseline information on demographics (age, gender, education, employment status, and contact details) and clinical data, which included comorbidities and current medications, and a section that allowed participants to record dates for upcoming clinical visits. However, the main purpose of this section was to encourage symptom monitoring on a daily basis, which included hemodynamic parameters, body weight, and any symptoms of fatigue, dizziness, or peripheral swelling, as guided by WHO-FC criteria [3]. This information was entered into a page labeled, Daily Assessment (Figure 1).

A real-time alert system was integrated to promptly identify abnormal readings entered into the Daily Assessment page. These alerts were transmitted to both the participant and the physicians. Details are provided in the section describing the symptom monitoring and alert system.

The fourth feature was a communication portal that supported interactions with other participants in the intervention group, which allowed them to exchange experiences, concerns, and emotional responses related to PH treatment. Users could choose to share information anonymously or under their real identities. This portal also provided participants access to support staff for consultations through secure messaging as well as direct communication with the research team for individualized support.

The third feature of the platform included patient education materials, which offered curated resources, including links to web-based videos developed by cardiologists and nurses. These resources addressed self-care strategies, medication guidance, emergency response protocols, nutrition, stress management, psychological support, and exercise techniques for PH management.

#### Symptom Monitoring and Alerts

When a participant recorded any measurement outside the predefined normal range, the alert system was automatically triggered. These alerts were determined by the 2022 ESC Guidelines, the 2018 Taiwan Society of Cardiology (TSOC) guidelines (specifically developed for Taiwanese patients) [3,28], and the expertise of the clinical research team regarding vital signs monitoring protocols. These references informed both the monitored items and the alert thresholds to ensure precise data collection and interpretation. Alarm levels were color-coded as high (red), raised (yellow), or normal (green) (Multimedia Appendix 2). For example, an SpO₂ reading ≤79% (red alarm) prompted the participant to rest and initiate home oxygen therapy for 5‐10 minutes. This would also automatically trigger a mobile app notification, prompting the research team to contact the participant within 30 minutes. Persistent severe symptoms necessitated immediate evaluation by the ED, with follow-up conducted by the research team. Additionally, if daily blood pressure data were not entered by 6 PM, automated reminder emails were sent to both the participant (or their caregiver) and the study team. The platform’s database was backed up daily at 11 PM to ensure data security.

#### Adherence and Technical Support

Participant adherence was automatically tracked via platform activity logs, with adherence defined as successful daily recording of symptoms and health parameters. The average rate of daily adherence and platform usage statistics was monitored throughout the intervention. Any technical issues encountered, such as login failures, data entry problems, or navigation difficulties, were logged and addressed promptly by the technical support team to minimize barriers to participation. Technical support was available throughout the study period via phone or web-based consultation. Participants continued daily self-monitoring for 6 months, supported by the educational resources and ongoing interactions available via the platform. Physicians and nurses regularly reviewed submitted data, provided timely feedback, adjusted medications as necessary, and offered additional clinical guidance during scheduled monthly visits or as indicated by the platform’s alert system.

#### Usual Standard Care

Usual standard care was received by the control group, which included regular outpatient follow-up clinic visits every 4 weeks with the treating physician of each participant. Treatment was individualized according to the clinical status of the participant, which is standard practice at our center. During these visits, physicians evaluated whether medication adjustments were needed, titrated PH-specific therapies as clinically indicated, and provided lifestyle counseling on topics such as diet, physical activity, medication adherence, and symptom management. Counseling was delivered verbally, which was standardized across all participants in the control group and supported by standardized educational pamphlets routinely used in our clinic to ensure consistency. If participants required additional advice or support between scheduled visits, telephone consultations were available as per usual clinical practice.

#### Measurements

##### Baseline Measures

All variables were assessed following enrollment (baseline, T=0) prior to randomization. Baseline measures for demographic and clinical characteristics, including age, gender, education, marital status, type of PH, and use of PH-targeted medications. The WHO-FC classification was used to categorize PH patients according to their symptom severity. The baseline assessment for the primary outcome of symptom interference was obtained from participants following enrollment using the symptom interference scale, described below. Baseline measures for secondary outcomes for physical activity (the 6MWT) were assessed by study-trained personnel after randomization, and echocardiographic measurements were collected from participants’ medical charts. Blood samples were collected from participants for measuring levels of NT-pro-BNP prior to randomization.

##### Symptom Interference

Symptom interference was measured with the 17-item self-report Pulmonary Arterial Hypertension Symptom Interference Scale (PAHSIS) [29], which assesses the impact of symptoms of PH on the daily life of a patient. Each item is a question about how much a symptom has affected the participant over the previous month. Symptoms include shortness of breath, fatigue, and sleep disturbance, which are scored from 0 (no interference) to 10 (extremely interfering). The total score possible ranges from 0 to 170; higher scores indicate greater interference with daily life due to symptoms of PH. The scale has been shown to be a valid, reliable scale for adult patients with PH, with a Cronbach α of .91 [29]. With permission from the original authors, the scale was translated into Taiwanese for our study by bilingual researchers using the forward- and back-translation method outlined by Brislin et al [30] and followed methods detailed by Cha et al [31] to ensure cultural equivalence. The Cronbach α for the Taiwanese translation of the scale was 0.86.

##### Six-Minute Walk Test and Dyspnea Assessment

The 6MWT was conducted by skilled research nurses specialized in PH following standardized American Thoracic Society (ATS) guidelines [32]. Participants were instructed to walk as far as possible within 6 minutes along an unobstructed, hard, flat, 30-meter corridor at their maximum comfortable pace [32]. Use of assistive walking devices was permitted if required. During the test, participants were closely monitored by trained PH research nurses, whose primary aim was to encourage maximal walking distance within the allocated time. Participants experiencing any discomfort were allowed to stop and rest at any point. Immediately after completion, the total distance walked and heart rate were recorded. The 6MWT is a widely accepted, simple, cost-effective, and reproducible tool commonly used to evaluate functional exercise capacity in patients with chronic cardiopulmonary conditions [33].

Post-test dyspnea severity was measured immediately using the Modified Borg Dyspnea Scale, a numerical rating scale ranging from 0 (representing no dyspnea at all) to 10 (representing maximal dyspnea). Each numerical rating is associated with verbal descriptors reflecting the intensity of dyspnea, such as mild, moderate, severe, or maximal effort [34]. The Borg scale is widely used due to its simplicity, reliability, and validity for evaluating perceived breathlessness in diverse patient populations [34].

##### TAPSE/sPAP and NT-Pro-BNP

The ratio of the tricuspid annular plane systolic excursion and systolic pulmonary artery pressure (TAPSE/sPAP) is a noninvasive, reproducible echocardiographic marker of right ventricular–pulmonary arterial (RV-PA) coupling, which can be used for PAH risk assessment [3]. Previous studies have demonstrated prognostic value in both patients with heart failure and PH [35]. The TAPSE/sPAP ratio was determined with the Philips iPQ7 system (Philips GmbH, Hamburg, Germany). A TAPSE/sPAP ratio <0.55 mm/mmHg is considered suggestive of PH; values ≤0.32 mm/mmHg are associated with an increased risk of all-cause mortality and are incorporated into current risk stratification models [3].

The structural and functional changes in RV-PA coupling lead to myocardial strain and hypertrophy, which stimulate the release of natriuretic peptides, particularly brain natriuretic peptide (BNP) and N-terminal pro-BNP, which are secreted by cardiomyocytes in response to increased ventricular wall stress [36]. The measurement of NT-pro-BNP thus provides complementary biochemical evidence of right ventricular overload and dysfunction in patients with PH. Blood samples for NT-pro-BNP measurement were collected at 4 time points: baseline (prior to intervention) and at 3, 6, and 9 months after enrollment, coinciding with each scheduled study assessment. All samples were analyzed at the hospital’s central laboratory using standardized procedures.

##### Emergency Department Readmission Events

Following enrollment in the study, we monitored participants for ED readmission events over a 9-month observation period. Data regarding ED visits were systematically collected through regular chart reviews and patient self-reports. Each readmission event was verified and documented, including the date, cause, duration of stay, and relevant clinical interventions. ED readmission rates served as a key secondary outcome to assess the effectiveness of our intervention in managing symptom exacerbations and preventing acute deterioration in patients with pre-capillary PH.

### Data Collection

**Figure 2.**
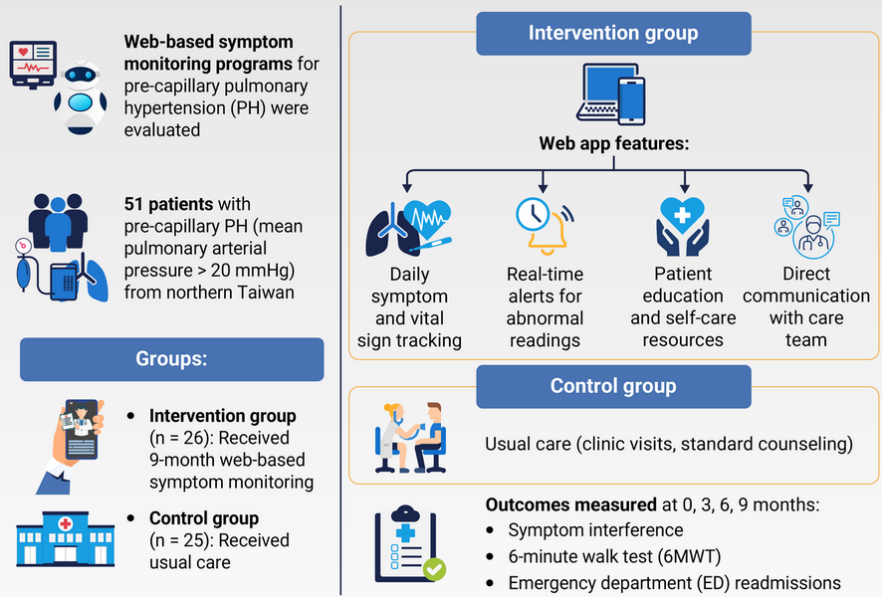
Overview of study design and outcomes.

Data were collected between February 2023 and August 2024. Baseline data for demographic and clinical characteristics and outcome variables were collected at enrollment prior to randomization (T0). Primary and secondary outcomes were also collected at completion of the intervention (3 mo following enrollment, T1), at 6 months (immediately after completion of the intervention, T2), and at 9 months (T3), corresponding to 3 months after the intervention had ended. Outcome assessors were blinded to group assignments and collected 6MWT distances and symptom interference scores without knowledge of intervention status to minimize measurement bias. An overview of the study design, participants, and outcome assessments used to evaluate the effects of the web-based monitoring program (Figure 2).

### Data Analysis

Data were analyzed using IBM SPSS (version 26.0; IBM Corp). Demographic and clinical characteristics of the 2 groups were examined using means and standard deviations (SD) for continuous variables and frequencies (n) and percentages (%) for categorical variables. Independent t-tests and one-way analysis of variance (ANOVA) assessed the homogeneity of characteristics between the groups. To evaluate the impact of the telemedicine program, we compared outcomes for participants in the experimental group with the control group using generalized estimating equations (GEE) [37,38]. Primary outcome measures included scores on the symptom interference score. Secondary outcomes included 6MWT, Borg scores, hemodynamic data (TAPSE/sPAP and NT-pro-BNP levels), and ED readmission events. The significance threshold for all analyses was set at *P*<.05 for 2-tailed *t*-tests. All dependent variables with missing data in the GEE conformed to the assumption Littles’ test of Missing Completely at Random (MCAR) [39]: *χ*^2^=62.397, DF =56, *P*=.25.

## Results

### Baseline Participant Characteristics

**Figure 3.**
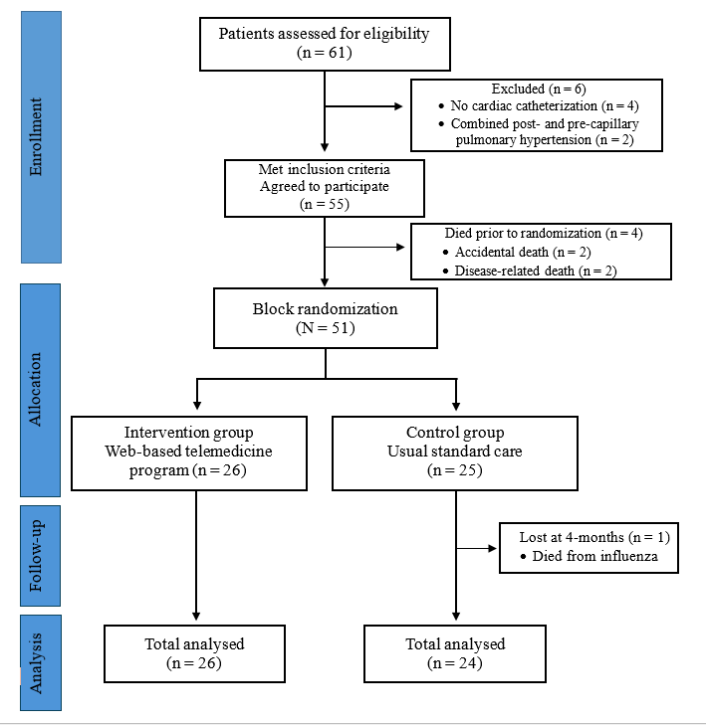
Flow diagram of the study.

A total of 61 patients were screened for inclusion in the study; 4 patients were excluded because they had not undergone cardiac catheterization, and 2 patients were diagnosed with combined post- and pre-capillary PH. Of the remaining 55 patients, 4 died before the start of the study due to accidental causes (n=2) and progressive disease (n=2). During the first 4 months of the study, 1 participant in the control group died of influenza. Figure 3 shows the CONSORT diagram outlining the flow of participants from patient selection, randomization, and analysis; the CONSORT checklist is included as Checklist 1.

The demographic and clinical characteristics of the 51 participants and differences between the 2 groups are shown in Table 1. The mean age was 59.6 (SD 13.6) years, most were female (74.5%, 38/51), and 52.9% (27/51) were married. The mean duration of PH was 3.38 (SD 2.55) years; 33.4% of participants received 2-3 PH-specific medications. Slightly more than half of all participants (52.9%, 27/51) were classified with Class II disease severity. The most common diagnoses of PAH were connective tissue disease-associated PAH (39.2%, 20/51) and idiopathic PAH (27.5%, 14/51). Baseline measures on the symptom interference were 27.25 (SD 20.81) and 6MWT were 365.68 (20.9) meters. There were no significant differences in demographic or clinical characteristics, or baseline scores for primary or secondary outcomes.

**Table 1.** Baseline demographics, clinical characteristics, and scale scores for all participants and differences between the intervention group and control group (N=51).

Variables		Groups		*P* value
	Participants (N=51)	Intervention (n=26)	Control (n=25)	
Age (years), mean (SD)^a^	59.60 (13.60)	57.69 (13.33)	61.60 (13.86)	.31
Gender (female), n (%)	38 (74.5)	20 (76.9)	18 (72.0)	.46
Married, n (%)	27 (52.9)	13 (25.5)	14 (27.4)	.20
Education, n (%)				.25
Less than junior high school	23 (45.1)	10 (19.6)	13 (25.5)	
High School and above	28 (54.9)	16 (31.4)	12 (23.5)	
Duration of PH^b^ (years), mean (SD)	3.38 (2.55)	3.45 (3.0)	3.31 (2.03)	.84
BMI (kg/m^2^), mean (SD)		22.84 (5.04)	22.05 (4.44)	.55
Type of PAH^c^, n (%)				.89
Idiopathic or heritable	14 (27.5)	8 (15.7)	6 (11.8)	
Congenital heart disease-associated	9 (17.6)	4 (7.8)	5 (9.8)	
Connective tissue disease-associated	20 (39.2)	11 (21.6)	9 (17.6)	
Other types of PAH	3 (5.9)	1 (2.0)	2 (3.9)	
Chronic thromboembolic PH	5 (9.8)	2 (3.9)	3 (5.9)	
WHO-FC^d^, n (%)				.78
Class I	15 (29.4)	7 (13.7)	8 (15.7)	
Class II	27 (52.9)	14 (27.5)	13(25.5)	
Class III	8 (15.7)	4 (7.8)	4 (7.8)	
Class IV	1 (2.0)	1 (3.8)	0 (0.0)	
PH medication, n (%)				
Phosphodiesterase 5-inhibitors	40 (78.4)	23 (45.1)	17 (33.3)	.09
Endothelin receptor antagonists	18 (35.3)	9 (17.6)	9 (17.6)	>.99
Soluble guanylate cycle stimulator	7 (13.7)	3 (5.9)	4 (7.8)	.70
Prostacyclin	2 (3.9)	1 (2.0)	1 (2.0)	>.99
IP receptor agonist	4 (7.8)	3 (5.9)	1 (2.0)	.61
Right heart catheterization, mean (SD)				
mPAP^e^ (mmHg)	37.7 (12.6)	38.76 (3.91)	36.68 (11.40)	.55
Pulmonary vascular resistance (WU^f^)	7.9 (5.9)	8.62 (7.05)	7.26 (4.69)	.42
Right atrial pressure (mmHg)	7.7 (5.3)	7.04 (5.95)	8.36 (4.79)	.39
Pulmonary arterial wedge pressure (mmHg)	7.9 (5.1)	7.16 (5.35)	8.31 (4.81)	.43
Cardiac output (L/min)	3.8 (1.0)	3.81 (1.12)	3.79 (1.06)	.90
Cardiac index (L/min/m^2^)	2.3 (0.6)	2.24 (0.68)	2.29 (0.60)	.41
Baseline outcomes, mean (SD)				
Primary outcome				
Symptom interference^g^ (PAHSIS)	27.25 (20.81)	25.53 (4.5)	29.4 (3.69)	.98
Secondary outcomes				
6MWT^h^, meters	365.68 (20.9)	355.22 (25.24)	76.14 (23.38)	.54
Borg Dyspnea Scale score (0‐10)	4.06 (2.00)	4.12 (2.1)	4.0 (2.1)	.84
TAPSE/sPAP^i^ ratio (mm/mmHg)	0.36 (0.21)	0.36 (0.20)	0.35 (0.22)	.93
NT-pro-BNP^j^ (pg/mL)	919.0 (1453.5)	732.5 (1296.1)	1130.3 (1616.1)	.42

a SD: standard deviation.

b PH: pulmonary hypertension.

c PAH: pulmonary arterial hypertension.

d WHO-FC: World Health Organization functional classification for severity of pulmonary hypertension.

e mPAP: mean pulmonary arterial pressure.

f WU: Wood unit.

g symptom interference=Pulmonary Arterial Hypertension Symptom Interference Scale.

h 6MWT: 6-min walking test.

i TAPSE/sPAP ratio: tricuspid annular plane systolic excursion/systolic pulmonary artery pressure ratio.

j NT-pro-BNP: N-terminal pro-brain natriuretic peptide.

### Platform Engagement and Alert System Utilization

Overall, participants in the intervention group showed good adherence to daily monitoring of symptoms, with an average daily compliance rate of 85%. Among the 26 intervention participants, 20 (76.9%) consistently recorded data daily throughout the 6-month intervention period, while 6 participants (23.1%) had intermittent compliance, with recording adherence ranging from 50% to 75% of days. Technical difficulties were infrequent, reported by only 4 participants (15.4%), and were promptly resolved by providing additional platform guidance or technical assistance from the research team. Detailed adherence data, including types and frequency of technical issues encountered, are summarized in Multimedia Appendix 3.

The real-time alert system was activated a total of 204 times during the intervention period. Among these alerts, approximately 76% were classified as yellow-level alerts indicating moderate clinical concerns, which prompted routine follow-ups and timely remote clinical advice from our health care team within approximately 24 hours. The remaining 24% were red-level alerts signaling more urgent clinical issues. These alerts triggered immediate interventions, including direct telephone consultations and tailored medical recommendations. In 13 cases, the severity of symptoms necessitated emergency medical interventions in hospital settings.

### Effect of the Intervention on Symptom Interference

**Figure 4.**
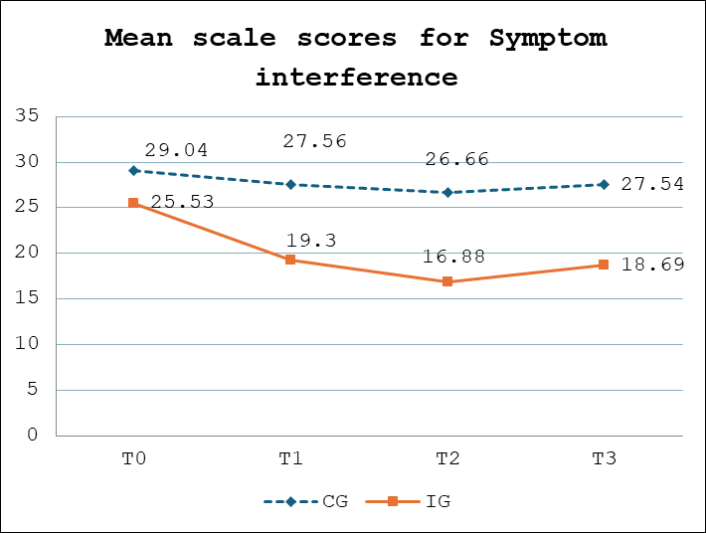
Differences in mean scale scores for the primary outcome of symptom interference at 3 (**T1**), 6 (**T2**), and 9 months (**T3**) compared with baseline measures (**T0**) for the intervention group (IG) compared with the control group (CG).  .

Examination of the effect of web-based symptom monitoring on the primary outcome of symptom interference using GEE analysis indicated that the web-based symptom monitoring program resulted in a greater reduction in symptom interference for participants in the intervention group compared with those in the control group. Specifically, mean scores on the PAHSIS for the intervention group improved significantly compared with scores for the control group at 3 months (*β*=−4.75; 95% CI −9.21 to −0.28; *P*=.03), and 6 months (*β*=−6.70; 95% CI −13.05 to −0.36; *P*=.03). In addition, mean scores on the PAHSIS at 9 months (3 mo following completion of the program for the intervention group), the significant reduction in scores from baseline for the intervention group compared with the control group was maintained (*β*=−5.43; 95% CI −12.5 to −0.29; *P*=.03) (Table 2 and Figure 4).

**Table 2.** Generalized estimating equation analysis of the effect of the intervention on the primary outcome of the symptom interference (n=51).

Outcome	β^a^	SE^b^	95% CI	Chi-square *(df)*	*P* value
Symptom interference^c^, PAHSIS					
Group (intervention)^d^	−3.50	5.72	−14.73 to 7.72	0.37 (1)	.54
Time^e^					
3 months (T1)	−1.48	1.51	−4.45 to 1.49	0.95 (1)	.32
6 months (T2)	−1.94	2.03	−5.93 to 2.03	0.91 (1)	.33
9 months (T3)	−1.41	1.77	−4.89 to 2.07	0.63 (1)	.42
Group (intervention)^d^×Time^e^					
3 months (T1)	−4.75	2.27	−9.21 to −0.28	4.35 (1)	.03
6 months (T2)	−6.70	3.23	−13.05 to −0.36	4.28 (1)	.03
9 months (T3)	−5.43	3.61	−12.5 to −0.29	4.27 (1)	.03

a β: estimate.

b SE: standard error.

c Symptom interference = scores on the Pulmonary Arterial Hypertension Symptom Interference scale.

d Reference: control group.

e Reference group: time (baseline, T0).

### Effect of the Intervention on the 6MWT and Dyspnea

**Figure 5.**
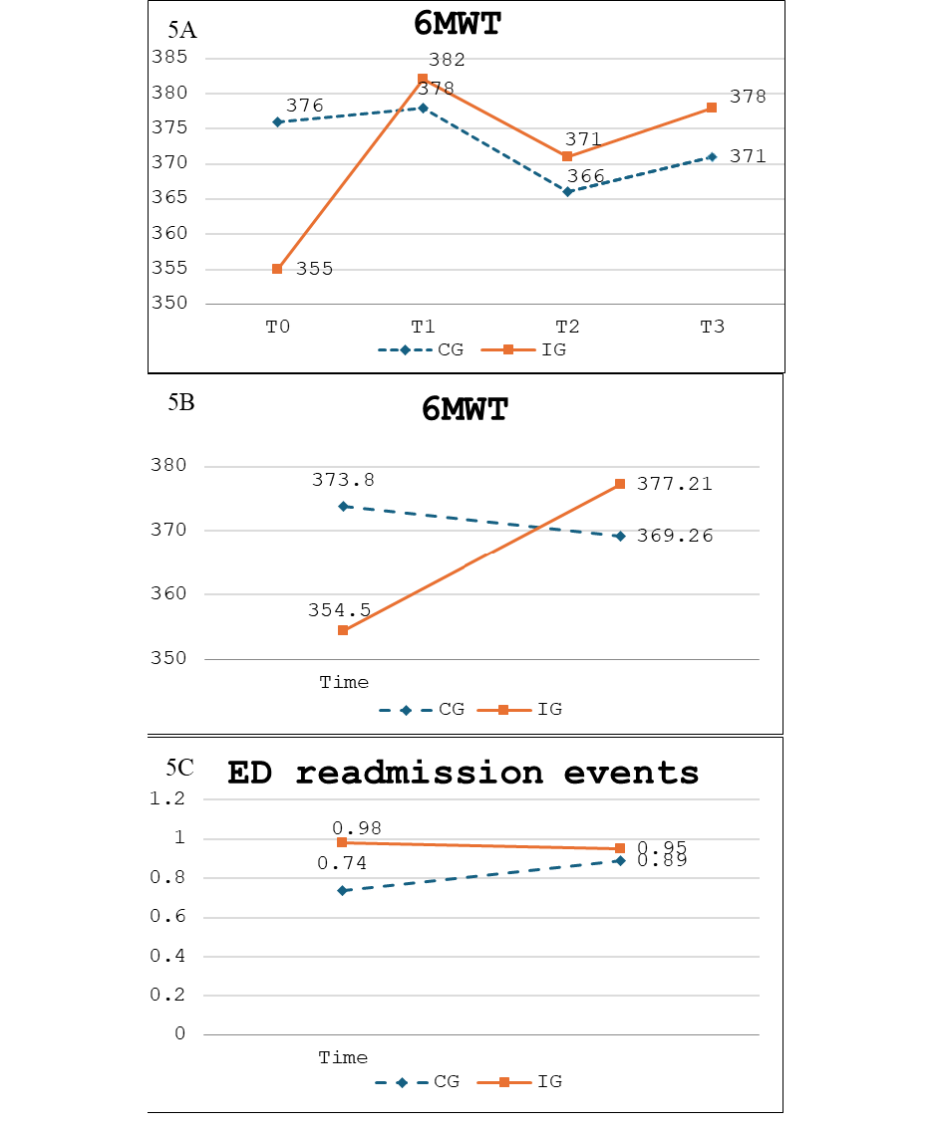
Significant effects of the intervention on secondary outcomes of the 6MWT and ED readmission events. (A) Differences from mean baseline scores (**T0**) at 3 months (**T1**), 6 months (**T2**), and 9 months (**T3**) for the intervention group (IG) compared with the control group (CG). (B) General estimating equation analysis of differences in predicted mean values on the 6MWT (Group × Time) for the IG compared with the CG. (C) Effect on the secondary outcome of emergency department (ED) readmission events: general estimating equation analysis of differences in predicted mean values (Group × Time) for the IG compared with the CG.

GEE analysis showed significant differences from baseline in the secondary outcome of the 6MWT between groups. Compared with the control group, participants in the intervention group had a significantly greater increase from baseline in distance at 3 months (*β*=25.20; 95% CI 0.63 to 49.78; *P*=.044) and 6 months (*β*=29.12; 95% CI 2.63 to 55.61; *P*=.031). In addition, when distances from baseline to 9 months were examined, compared with the control group, the increase in distance for the intervention group (3 mo following completion of the intervention) was similar to measures at 3 and 6 months (*β*=29.67; 95% CI 3.15 to 56.19; *P*=.02), suggesting improvements on the 6MWT were sustained over the 3 months following completion of the intervention. (Figure 5A and Table 3). Additionally, analysis demonstrated the distance on the 6MWT increased by 9.8 meters every 3 months for the intervention group compared to the controls over the 9 months of the study period (*β*=9.81; 95% CI 0.93 to 18.70; *P*=.03) and (Figure 5B and Table 3).

The mean score on the Borg Scale also decreased significantly for the intervention group compared with controls at 3 months (*β*=−1.0; 95%CI −1.76 to −0.237; *P*=.01) and 6 months (*β*=−1.04; 95% CI −2.05 to −0.044; *P*=.04) (Table 3).

**Table 3.** Generalized estimating equation analysis demonstrating significant effects of the intervention on secondary outcome (n=51).

Variable	β^a^	SE^b^	95% CI	Chi-square *(df)*	*P* value
6MWT^c^					
Group (intervention)^d^	−20.92	33.71	−86.99 to 45.15	0.38 (1)	.53
Time^e^					
3 months (T1)	1.98	9.34	−16.33 to 20.29	0.04 (1)	.83
6 months (T2)	−12.38	7.88	−27.83 to 3.07	2.46 (1)	.11
9 months (T3)	−6.87	8.89	−24.30 to 10.55	0.59 (1)	.44
Group (intervention)^d^×Time^e^					
3 months (T1)	25.20	12.53	0.63 to 49.78	4.80 (1)	.04
6 months (T2)	29.12	13.51	2.63 to 55.61	4.64 (1)	.03
9 months (T3)	29.67	13.53	3.15 to 56.19	4.04 (1)	.02
Change in predicted mean value					
Group (intervention)^d^	−30.03	34.05	−96.78 to 36.72	0.77 (1)	.37
Time (slope)^f^	−2.22	2.99	−8.10 to 3.64	0.55 (1)	.45
Group (intervention) ^d^×Time (slope)^g^	9.81	4.53	0.93 to 18.70	4.69 (1)	.03
Borg Dyspnea Scale score					
Group (intervention)^d^	0.12	0.58	−1.03 to 1.27	0.04 (1)	.83
Time^e^					
3 months (T1)	−0.16	0.23	−0.63 to 0.31	0.45 (1)	.50
6 months (T2)	−0.19	0.32	−0.83 to 0.44	0.36 (1)	.54
9 months (T3)	−1.18	0.50	−2.18 to −0.19	5.51 (1)	.01
Group (intervention) ^d^× Time^e^					
3 months (T1)	−1.0	0.38	−1.76 to −0.237	6.59 (1)	.01
6 months (T2)	−1.04	0.51	−2.05 to −0.044	4.18 (1)	.04
9 months (T3)	−0.01	0.63	−1.26 to 1.237	0.00 (1)	.98

a B: estimate.

b SE: standard error.

c 6MWT: 6-min walking test.

d Reference: control group.

e Reference group: time (baseline, T0).

f Slope =change from T0 to T3.

g Slope =difference between the slope for the intervention group and the slope for the control group.

### Effects on Clinical Echocardiography and NT-Pro-BNP

We examined if there were effects on secondary outcomes for participants who received the web-based symptom monitoring program intervention compared with controls. There were no significant changes from baseline between the 2 groups for measures of TAPSE/sPAP ratio and the biomarker NT-pro-BNP at 3, 6, and 9 months (Multimedia Appendix 4).

### Effects on Emergency Department Readmissions Event

Finally, GEE analysis indicated that over the 9-month period, there was a significant decrease in ED readmission events for the intervention group compared with controls (*β*=−1.03; 95% CI −2.03 to −0.04; *P*=.04). Additionally, compared with controls, the likelihood of ED visits for the intervention group decreased by 65% every 3 months (OR=0.35; *P*=.04) (Table 4 and Figure 5C).

**Table 4.** Generalized estimating equation analysis demonstrating significant effects of the intervention on ED readmission events (n=51).

Change in ED^a^ readmission events outcome	ß^b^	SE^c^	95% CI	Chi-square *(df)*	*P* value
Group (intervention)^d^	3.93	1.12	1.72 to 6.14	50.98 (1)	<.001
Time (slope)^e^	0.53	0.37	−0.21 to 1.28	1.70 (1)	.15
Group (intervention)^d^x Time (slope)^f^	−1.03	0.50	−2.03 to −0.04	0.35 (1)	.04

a ED: emergency department.

b B: estimate.

c SE: standard error.

d Reference: control group.

e Reference group: slope of T1-T3.

f Slope =difference between the slope for the intervention group and the slope for the control group.

## Discussion

### Main Findings

This is the first randomized controlled trial to examine the effects of a web-based symptom monitoring program for patients with pre-capillary PH. The results demonstrated that the 6-month web-based symptom monitoring program reduced the number of disturbances participants experienced from symptom interference, increased the distance they were able to walk during the 6MWT, improved dyspnea scores, and reduced ED readmission rates. Importantly, the effect of the intervention on the outcome of the 6MWT was sustained 3 months after the completion of the program.

The web-based intervention allowed participants to remotely monitor their symptoms and provided guidance for improving their diet, reducing stress, managing depression, accessing remote web-based consultations, and exercising regularly on a daily basis. The significant sustained reduction in symptom interference experienced by the intervention group over 3 months after completion of the program compared with the control group suggests that providing training in monitoring important vital signs along with strategies and support for managing lifestyle should be part of routine care for patients with pre-capillary PH. The inclusion of a home system for alerting care providers about abnormal values allowed for medication adjustments and timely consultations, which may also have increased participants’ awareness of behaviors that resulted in poor disease management outcomes.

To further elucidate the potential mechanisms underlying the positive outcomes observed in our study, we examined the participants’ engagement with the web-based monitoring platform. Analysis of the platform usage data revealed that, on average, participants in the intervention group accessed the system approximately 5.2 times per week throughout the 6-month study period, with an average daily compliance rate for symptom monitoring of 85%. The platform features most frequently used by participants included symptom recording (95% utilization rate), patient education materials (87% utilization rate), and consultation portals (72% utilization rate).

We suggest that regular, individualized feedback provided through the alert system and direct patient-provider interactions substantially enhanced participants’ self-awareness and confidence in symptom management, thereby encouraging proactive health behaviors. These engagement-driven mechanisms likely contributed significantly to the observed improvements in symptom interference and the reduction in ED readmission events.

In addition, the intervention was successful in improving scores on the 6MWT and the sustained increase at 9 months suggested that the intervention helped participants establish self-monitoring habits and routine disease management. This outcome is in contrast to a study by Hemnes et al [23], who reported that, although a 12-week message-based intervention significantly increased step counts, there were no significant increases in distance on the 6 MWT in the intervention group compared to controls. In contrast, the mean distance traveled by the intervention group increased by 9.8 meters every 3 months compared to the control group. The sustained improvement in 6MWT distance observed in our intervention group has important clinical implications, potentially translating into meaningful enhancements in patients’ functional independence and quality of life.

Limited access to medical resources can prevent patients from taking an active role in their treatment, which can lead to feelings of helplessness [15,40]. Our program was guided by the increasing recognition that providing patients with self-management strategies can encourage them to actively engage in their care [41]. This web-based intervention program provided patients with easily accessible information on precapillary PH, which allowed them to make informed decisions and take action to manage their disease. The 2022 ESC/ERS PH guidelines endorse web-based self-monitoring as an effective management tool for PH [3], and our findings further reinforce its importance in controlling symptoms and improving patient mobility. The variability in symptoms of patients with PH highlights the need for integrated web-based interventions, as suggested by Gonzalez-Garcia et al [19] and Humbert et al [3]. These interventions are critical for tailoring treatment and monitoring strategies to individual patient needs and have the potential to transform standard PH management practices.

The significant reduction in symptom interference and dyspnea in the intervention group compared with the control group marks a pioneering finding in the PH population. This aligns with previous studies on patients with heart failure that reported similar improvements [42,43]. These improvements may be attributable to consultations and reminders provided by clinical professionals through the web-based program, which facilitated remote monitoring and early symptom detection, thereby improving patient management. The platform supports the daily monitoring of vital signs and provides remote feedback, including treatment recommendations from cardiologists.

Our alert system ensured close monitoring by researchers and physicians, enabling timely interventions such as adjusting medications (eg, diuretics) to prevent fluid overload and modifying targeted drugs to mitigate immediate life-threatening risks. One reason for frequent ED visits among patients with PH is that adjustments in medication dosage often destabilize BP and HR [7]. The continuous monitoring provided to participants in the intervention group may explain the reduction in ED readmission events that they experienced over the 9 months.

Limited medical resources can also prevent patients with PH from gaining a firm grasp of the impact of their disease and the need for treatment, which can prevent them from being receptive to advice on symptom monitoring, medication adherence, and lifestyle modifications [15]. Our platform addresses these challenges by providing participants with comprehensive remote care services to manage not only the disease but also their lifestyle. Participants had 24-hour access to educational resources, such as interactive videos and structured training modules, which demonstrated the relationships between symptoms and their disease while outlining effective management strategies. These materials were designed to improve patient adherence to prescribed medications, encourage physical activity, promote proper nutrition, emphasize the importance of vaccination, and provide guidance for weight management [3]. Additionally, emotional support from the nursing staff was designed to strengthen patient engagement and foster collaboration toward shared therapeutic goals. This comprehensive approach underscores the potential of web-based interventions to transform PH management practices.

We did not observe significant differences in the TAPSE/sPAP ratio or NT-pro-BNP levels between the groups. Notably, a recent meta-regression demonstrated that changes in mPAP, when measured invasively, have the highest explanatory power for functional improvement, a relationship that our noninvasive approach may not fully capture [44]. Our study relied on echocardiography rather than right-heart catheterization to evaluate the TAPSE/sPAP ratio, which may have contributed to the absence of significant between-group differences.

There are several possible explanations for these findings. Most participants in our study had a stable, less severe disease (27 classified as WHO-FC II), which may have limited the likelihood of observing substantial physiological changes over a 9-month period. In addition, noninvasive measures such as echocardiography and NT-proBNP may lack sensitivity in detecting subtle functional changes, especially in early-stage PH [45,46]. Moreover, medication regimens, including diuretics and PAH-targeted therapies, remained largely unchanged between the groups, suggesting that behavioral interventions alone, without pharmacological adjustments, may not be sufficient to induce measurable changes in right ventricular structure or function over a relatively short duration [23].

Importantly, it is also possible that the benefits of web-based interventions, such as improvements in patient-reported outcomes and reduced ED readmissions, may precede or even occur independently of detectable changes in cardiac structure or function, particularly among patients with milder disease. These findings underscore the need for future studies to identify the most responsive clinical parameters and clarify the temporal relationship between symptomatic improvements and objective physiological outcomes, ideally using more sensitive or longitudinal assessments, such as repeated RHC.

Finally, our findings showed that the intervention was associated with a 65% reduction in readmission rates every 3 months compared with the control group. Although this aligns with evidence from heart failure populations demonstrating reduced readmission and mortality through telemedicine interventions [47], disease-specific evidence for PH remains limited. Although our results provide promising preliminary support for web-based symptom monitoring in reducing ED readmissions among patients with pre-capillary PH, conclusions regarding mortality and long-term outcomes should be drawn cautiously. Larger, disease-specific trials are needed to confirm these benefits and clarify the long-term impact of digital health interventions on survival in PH.

### Implications and Future Research

Our results suggest that integrating web-based symptom monitoring interventions into the clinical care of patients with pre-capillary PH can significantly reduce ED readmissions and potentially lower mortality by enhancing patient engagement and at-home self-monitoring. While these findings provide important preliminary evidence of the effectiveness of the program, their generalizability must be considered cautiously, given our single-center design, relatively small sample size, and the demographic composition of our study population, which was predominantly female and largely limited to WHO Functional Class II severity in a single center in Taiwan. These factors may restrict the applicability of our findings to broader or more diverse populations with PH, geographical regions, and other health care systems. Although a post hoc power analysis confirmed adequate statistical power (power >0.8, medium effect size), larger multicenter trials enrolling a more heterogeneous cohort across different geographic regions and broader disease severities (including WHO Classes III and IV) are essential to validate and extend our results. In addition, long-term studies involving patients with poorer prognoses, such as those with connective tissue disease–associated PAH (CTD-PAH), are needed to explore the full potential of web-based interventions in managing PH [48]. Furthermore, future research should evaluate the impact of web-based symptom monitoring interventions in patients with more advanced disease severity as well as in multicenter, geographically diverse populations to enhance the generalizability of the findings. Incorporating more sensitive physiological endpoints such as repeated RHC measurements may also provide deeper insights into the mechanisms underlying the clinical benefits and clarify the impact of the intervention on disease progression.

### Limitations

Despite its strengths, this study had several limitations. First, most participants (52.9%) had WHO Functional Class II disease severity, which may limit the generalizability of our findings to a broader population of patients with pre-capillary PH. Although our post hoc statistical power analysis confirmed adequate statistical power (>0.8), the predominance of Class II participants restricted the extrapolation of results to patients with more advanced or varied disease severity. Second, owing to the limited number of eligible patients and the death of 4 patients prior to randomization, only 51 participants were enrolled by the end of the study period. The small sample size may have affected the statistical power and generalizability of the findings. Future research should therefore involve larger sample sizes and multiple clinical centers, recruiting patients from a more diverse spectrum of disease severities (such as WHO Classes III and IV) to validate and extend our findings. Third, measuring hemodynamic parameters can be challenging because these parameters may be influenced by variables such as physical activity levels and ED readmission events. Our use of echocardiography instead of more precise techniques such as right heart catheterization may have resulted in less accurate assessments of these critical clinical variables. Future studies should use repeated RHC assessments or other sensitive measurement methods to better capture subtle physiological changes over time.

### Conclusions

Compared with participants who received usual care, those who received the 6-month web-based symptom monitoring program experienced a significant reduction in symptom interference and dyspnea and improvements in physical ability as demonstrated by the increased distance on the 6MWT. The reduced symptom interference and exercise capacity were sustained for 3 months after completing the program, suggesting that the program is a viable means of improving disease self-management for patients with pre-capillary PH. The lower ED readmission events for participants in the intervention group compared with controls suggest that the program may also be a means of reducing medical costs associated with emergency care. Future research with larger sample sizes is needed to evaluate the impact of the intervention on clinical outcomes, particularly by measuring hemodynamic parameters using RHC across a geographically diverse population of patients with PH.

## Supplementary material

10.2196/76883 Multimedia Appendix 1 Screenshot of login to access the web-based monitoring system and an overview of the platform.

10.2196/76883 Multimedia Appendix 2 Alarm level and actions based on symptom monitoring measures.

10.2196/76883 Multimedia Appendix 3 Overview of web-based adherence to record keeping and website difficulties (n=26).

10.2196/76883 Multimedia Appendix 4 Generalized estimated equation analysis of changes from baseline in secondary outcomes of the TAPSE/sPAP ratio and NT-pro-BNP (N=51).

10.2196/76883 Checklist 1 CONSORT-eHEALTH checklist (V 1.6.1).

## Abbreviations

6MWT: 6-min walk test
CTEPH: chronic thromboembolic pulmonary hypertension
ED: emergency department
ESC/ERS: European Society of Cardiology/European Respiratory Society
GEEs: generalized estimating equations
mPAP: mean pulmonary arterial pressure
NT-pro-BNP: N-terminal pro-brain natriuretic peptide
PAH: pulmonary arterial hypertension
PAHSIS: Pulmonary Arterial Hypertension Symptom Interference Scale
PH: pulmonary hypertension
RHC: right heart catheterization
sPAP: systolic pulmonary arterial pressure
SpO2: peripheral oxygen saturation
TAPSE: tricuspid annular plane systolic excursion
TAPSE/sPAP ratio: tricuspid annular plane systolic excursion/systolic pulmonary arterial pressure ratio
WHO-FC: World Health Organization functional classification for severity of pulmonary hypertension.
