# Supplementary material for: Effects of Web-Based Symptom Monitoring Program on Symptom Interference, Physical Activity, and Emergency Department Readmissions in Patients With Pre-Capillary Pulmonary Hypertension: Randomized Controlled Trial
Source: J Med Internet Res. 2025 Sep 15;27:e76883. doi: 10.2196/76883 (PMC12440832; doi:10.2196/76883)

Multimedia Appendix 1. Screenshot of Login to access the Web-based Monitoring System and an overview of the platform.

Login page for participants in the intervention group


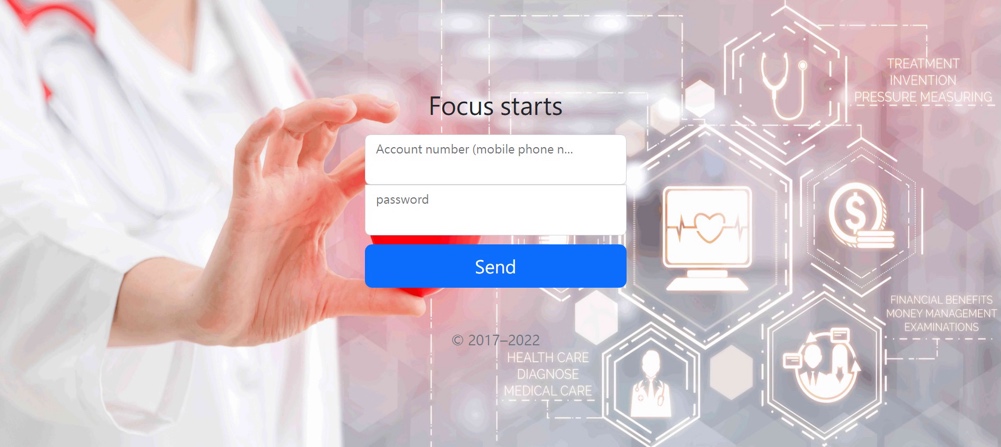


Web-based Monitoring Program


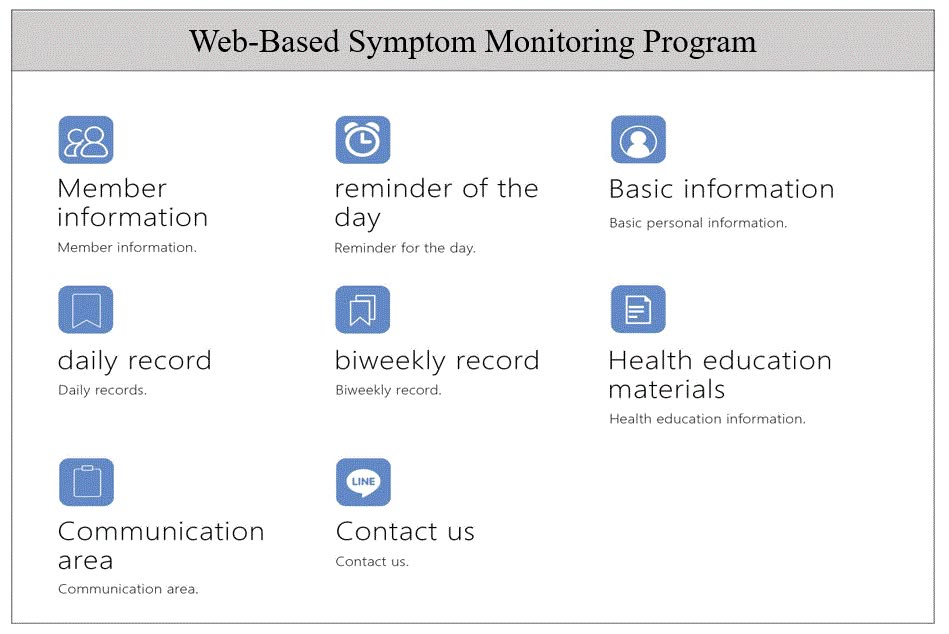


Web-based health education materials


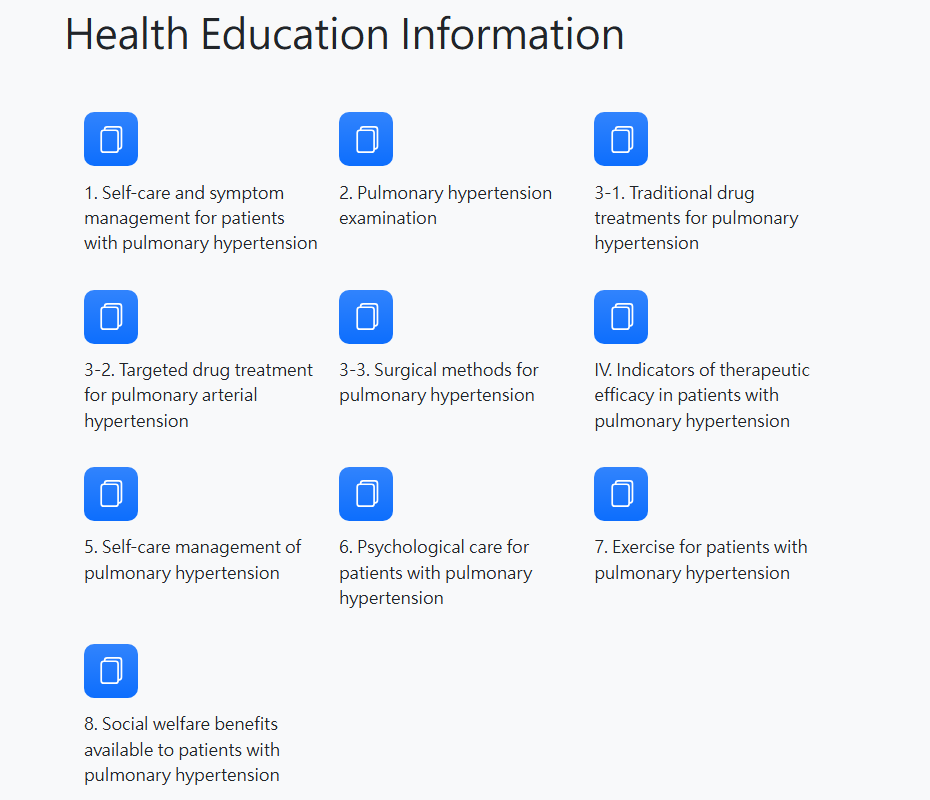

Supplement: Multimedia Appendix 1 [file jmir-v27-e76883-s001.docx]
