# Supplementary material for: Effects of Web-Based Symptom Monitoring Program on Symptom Interference, Physical Activity, and Emergency Department Readmissions in Patients With Pre-Capillary Pulmonary Hypertension: Randomized Controlled Trial
Source: J Med Internet Res. 2025 Sep 15;27:e76883. doi: 10.2196/76883 (PMC12440832; doi:10.2196/76883)
Supplement: Multimedia Appendix 2 [file jmir-v27-e76883-s002.docx]

**e-Appendix 2.** Alarm level and actions based on symptom monitoring measures

| Color | Level | Measurement | Action |
| --- | --- | --- | --- |
| Red | High | SBP ≤79 mmHg  **OR**  DBP ≤49 mmHg  **OR**  HR ≤ 49 for ≥121 minutes  **OR**  SpO2 ≤ 79%  **OR**  Borg Scale score = 9-10  **OR**  Body weight ≥ 2kg | - If you have just finished exercising, sit down and rest for 5-10 minutes, take deep breaths; if you experience difficulty breathing, use oxygen and continue to monitor your oxygen levels. - If your oxygen levels remain ≤ 79% despite using oxygen, and you feel chest tightness, dizziness, or palpitations, please go to the nearest emergency room immediately. - Researchers will contact you within 30 minutes to 1 hour. |
| Yellow | Raised | SBP 80-89mmHg  **OR**  DBP 50-59 mmHg  **OR**  HR ≤ 50-60 for ≥110-120 min  **OR**  SpO2 80-89%  **OR**  Borg Scale score = 6-8  **OR**  Body weight ≥ 1kg | - If you have just finished exercising, sit down and rest for 5-10 minutes, take deep breaths; if you experience difficulty breathing, use oxygen and continue to monitor your oxygen levels. - Ensure that you have taken your medications as prescribed today, and check if you have missed any doses. - Review whether you have recently consumed excessive fluids or sodium. - Check if you have recently experienced symptoms of a cold or other infections. - If you still feel unwell, such as experiencing palpitations, chest tightness, or difficulty breathing, please use the online contact system to reach us, or visit the nearest emergency room for treatment. - Researchers will contact you within 24 hours. |
| Green | Normal | SBP 90-140mmHg  **OR**  DBP ≥60 mmHg  **OR**  HR 60-109  **OR**  SpO2 90-100%  **OR**  Borg Scale score = 0-5  **OR**  Body weight < 1kg | - Your physiological monitoring values and symptoms are within the normal range. - Please continue to measure and upload your physiological data daily, allowing the research team to monitor your symptoms. - Ensure you take your medications regularly and check if any of your medications are causing uncomfortable side effects. - Maintain a healthy lifestyle, limit daily fluid intake to less than 1500 mL, and take your medications on time. |
