# Supplementary material for: Effects of Web-Based Symptom Monitoring Program on Symptom Interference, Physical Activity, and Emergency Department Readmissions in Patients With Pre-Capillary Pulmonary Hypertension: Randomized Controlled Trial
Source: J Med Internet Res. 2025 Sep 15;27:e76883. doi: 10.2196/76883 (PMC12440832; doi:10.2196/76883)
Supplement: Multimedia Appendix 3 [file jmir-v27-e76883-s003.docx]

Multimedia Appendix 4: Overview of Web-based Adherence to record keeping and web-site difficulties (n = 26)

| Variable | n (%) | Mean ± SD |
| --- | --- | --- |
| Adherence measure |  |  |
| Compliance with daily recordings |  |  |
| Overall compliance rate |  | 85.0% ± 10.5% |
| ≥ 90% | 20 (76.9%) |  |
| 50%-75% | 6 (23.1%) |  |
| Frequency of weekly login, days/week |  | 5.2 ± 1.1 |
| Technical difficulties |  |  |
| Number of difficulties | 4 (15.4%) |  |
| Types of technical difficulties |  |  |
| Difficulty logging into the platform | 2 (7.7%) |  |
| Data entry or saving issues | 1 (3.8%) |  |
| General usability questions | 1 (3.8%) |  |
| Mean time to resolve technical issues, days |  | 1.3 ± 0.5 |
