# Supplementary material for: Effects of Web-Based Symptom Monitoring Program on Symptom Interference, Physical Activity, and Emergency Department Readmissions in Patients With Pre-Capillary Pulmonary Hypertension: Randomized Controlled Trial
Source: J Med Internet Res. 2025 Sep 15;27:e76883. doi: 10.2196/76883 (PMC12440832; doi:10.2196/76883)
Supplement: Multimedia Appendix 4 [file jmir-v27-e76883-s004.docx]

**Multimedia Appendix 5.** Generalized estimated equation analysis of changes from baseline in secondary outcomes of the TAPSE/sPAP ratio and NT-pro-BNP (N = 51)

| Variable | B | SE | 95% CI | *χ*2 | *P* |
| --- | --- | --- | --- | --- | --- |
| TAPSE/sPAP ratio |  |  |  |  |  |
| Group (Intervention)^a^ | 0.11 | 0.06 | -0.11 to 0.13 | 0.32 | .85 |
| Time ^b^ |  |  |  |  |  |
| 3 months (T1) | -0.002 | 0.044 | -0.08 to 0.09 | 0.02 | .96 |
| 6 months (T2) | -0.049 | 0.055 | -0.16 to 0.06 | 0.78 | .37 |
| 9 months (T3) | -0.052 | 0.048 | -0.15 to 0.04 | 1.14 | .28 |
| Group (Intervention)^a^ x Time ^b^ |  |  |  |  |  |
| 3 months (T1) | 0.006 | 0.498 | -0.09 to 0.10 | 0.01 | .91 |
| 6 months (T2) | 0.052 | 0.062 | -0.07 to 0.18 | 0.68 | .40 |
| 9 months (T3) | 0.085 | 0.059 | -0.03 to 0.20 | 2.00 | .15 |
| NT-pro-BNP |  |  |  |  |  |
| Group (Intervention)^a^ | -1809 | 1438.9 | -4629.3 to 1011.2 | 1.58 | .20 |
| Time ^b^ |  |  |  |  |  |
| 3 months (T1) | -1198 | 1214.4 | -3578.9 to 1081.7 | 0.94 | .32 |
| 6 months (T2) | -1330 | 1168.5 | -3621.0 to 959.5 | 1.29 | .25 |
| 9 months (T3) | -283.8 | 320.2 | -911.4 to 343.8 | 0.78 | .37 |
| Group (Intervention)^a^ x Time ^b^ |  |  |  |  |  |
| 3 months (T1) | 1071 | 1216.6 | -1313.5 to 3455.6 | 0.77 | .37 |
| 6 months (T2) | 1290 | 1174.4 | -1011.7 to 3592.1 | 1.20 | .27 |
| 9 months (T3) | 603.2 | 444.0 | -267.1 to 1473.6 | 1.84 | .17 |

B = estimate; SE = standard error; TAPSE/sPAP ratio = tricuspid annular plane systolic excursion/systolic pulmonary arterial pressure ratio; NT-pro-BNP = N-terminal pro-brain natriuretic peptide

^a^ Reference: Control group

^b^ Reference group: Time (baseline, T0)
